# Prophylactic Breast Bud Radiotherapy for Patients Taking Bicalutamide: Should This Still Be Practised for Patients with Prostate Cancer?

**DOI:** 10.1155/2012/239269

**Published:** 2012-08-28

**Authors:** R. Lewis, A. Cassoni, H. Payne

**Affiliations:** Department of Clinical Oncology, University College Hospital London, London NW1 2PG, UK

## Abstract

Prophylactic breast bud radiotherapy is used to prevent gynaecomastia and mastalgia in patients with prostate cancer who are being treated with antiandrogen and oestrogen therapy. Here a case is presented of a patient who developed soft-tissue sarcoma of the breast subsequent to breast bud radiotherapy prior to bicalutamide hormone treatment. Bicalutamide is often prescribed for younger men in the adjuvant setting or as monotherapy for locally advanced disease. The data regarding the efficacy of prophylactic breast bud radiotherapy is reviewed, and it is proposed that alternative therapies should be considered such as tamoxifen.

## 1. Introduction

Current National Institute of Clinical Excellence (NICE) Guidelines (February 2008) state that “men starting long-term bicalutamide 150 mg monotherapy (>6 months) should receive prophylactic radiotherapy to both breast buds within the first month of treatment. A single fraction of 8 Gy using orthovoltage or electron beam radiotherapy is recommended” [1].

Prophylactic breast irradiation is commonly used for men prior to commencing stilboestrol therapy for advanced prostate cancer in the palliative setting. However, bicalutamide 150 mg is licenced for men with locally advanced disease. It can be prescribed as monotherapy or as adjuvant treatment in combination with radical radiotherapy to the prostate or with salvage postoperative prostate bed radiotherapy. In these groups of patients, there are different therapy intentions and also a much longer anticipated survival to those being treated with stilboestrol for advanced metastatic castration-resistant prostate cancer. The use of prophylactic breast irradiation as an attempt to prevent gynaecomastia and mastalgia with bicalutamide 150 mg, therefore causes concern regarding the long-term effects of treating a benign condition with radiation especially with regards to the risk of long-term second malignancy in men who may otherwise be “cured” of or in remission from their prostate cancer. We would like to present case of a man who developed breast cancer as a result of preventative radiation treatment.

## 2. The Case

A man who is now 82 presented in 2001 with stage 3b, gleason grade 3 + 4 adenocarcinoma of the prostate. His PSA at diagnosis was 7.5. He was treated at another hospital with neoadjuvant bicalutamide 150 mg once daily for 3 months and then received radiotherapy to a dose of 55 Gy in 20 fractions to the prostate and seminal vesicles. He continued with bicalutamide 150 mg for 6 months in the adjuvant setting and during this time was reported to have developed breast pain and gynaecomastia. He had an initial response to combination therapy with a PSA nadir of 2. However, in 2003, he had a PSA relapse and was recommenced on bicalutamide 150 mg daily. In view of the previous breast symptoms, he received prophylactic breast bud radiotherapy to a dose of 15 Gy in 3 fractions, delivered on alternate days with kv photons. His PSA began to rise again in 2010 and he started maximum androgen blockade with goserelin and bicalutamide 50 mg daily with a further good PSA response. In January 2011, 10 years after being diagnosed with prostate cancer and 8 years after prophylactic breast bud irradiation, he presented with a hard palpable mass in the right breast. On biopsy, this was found to be a high-grade Trojani grade 2 pleomorphic spindle cell sarcoma of the right breast. This was excised with adequate margins and he received no further adjuvant treatment. He remains well and active.

## 3. Secondary Malignancy after Radiotherapy

There is very little data on the risk of developing a second malignancy after prophylactic breast bud radiotherapy; however, there is more evidence for patients receiving higher doses of radiation for adjuvant treatment to the whole breast for breast cancer. For example, a cohort study of 5248 women treated in Italy indicated an increased relative risk of all second cancers combined following breast radiotherapy (1.22, 95% CI: 0.88 to 1.69). The increased relative risk appeared five or more years after radiotherapy and appeared to be highest amongst women treated after the menopause (1.61, 95% CI: 1.13 to 2.29) [2]. This confirms data from another larger-scale cohort study of 182, 057 patients from the USA SEER cancer registries who were 5-year survivors of locoregional breast cancer. They found the relative risk of developing a second malignancy in areas receiving high doses of radiotherapy was 1.45 (95% CI 1.33–1.58) [3].

These patients were receiving much higher doses of radiotherapy (50 Gy/25#) with megavoltage photons, and the conclusions of the research was that the risk of second malignancy was low compared with the benefit of adding adjuvant radiotherapy to the breast/chest wall in reducing disease recurrence. However, in prostate cancer patients, breast bud radiotherapy is being used as prophylaxis for a benign and often reversible condition with a theoretical risk of causing a malignancy.

## 4. Bicalutamide

Bicalutamide 150 mg is a nonsteroidal antiandrogen which can be used in locally advanced prostate cancer as an alternative to castration-based therapy with luteinising hormone-releasing hormone agonists (LHRHa). Bicalutamide 150 mg has some advantages over castration-based therapy in that it can maintain physical capacity and bone mineral density and reduces the risk of hot flushes and loss of sexual function. This is however at the expense of an increased risk of gynaecomastia and mastalgia. Bicalutamide 150 mg has hypergonadotropic effects, and androgens are aromatised in extragonadal tissues to 17B-oestrodiol, which induces the benign proliferation of breast tissues and causes gynaecomastia and associated breast pain during the proliferative phase. 

The potential toxicity advantages of bicalutamide 150 mg mean that it is often prescribed for younger men with locally advanced disease. There is evidence from the Early Prostate Cancer (EPC) programme for its efficacy as monotherapy and adjuvant to radical radiotherapy in this setting [4]. In the fourth and final 10-year analysis from this large prospective, randomised study of 8113 men, there was a significant improvement in progression-free survival in patients who received bicalutamide versus those who received placebo in addition to standard care (watch and wait, radiotherapy, radical prostatectomy) in the locally advanced subgroup. An overall survival benefit was shown for patients with locally advanced prostate cancer who received radiotherapy and adjuvant bicalutamide as opposed to placebo (70% versus 58%; HR = 0.65, *P* = 0.03). The EPC study reported that gynaecomastia and breast pain occurred in 66% and 73% of patients receiving bicalutamide 150 mg, respectively, and 16% of patients discontinued treatment because of this, highlighting the need for prophylaxis in some patients.

A more recent further potential indication for the use of bicalutamide 150 mg is in combination with adjuvant or salvage prostate bed irradiation for men with high-risk factors after radical prostatectomy. This is supported by preliminary data from the RTOG 96-01 trial [5]; a phase III study, randomising 771 men with pT2-3N0 M0 disease and a rising PSA after prostatectomy to bicalutamide 150 mg daily or placebo for two years in combination with salvage prostate bed radiotherapy. With median followup 7.1 years, the overall survival was 91% for the radiotherapy and bicalutamide group and 86% for those treated with radiotherapy alone. This did not show a significant difference due to the fact that too few primary endpoint events have occurred to allow a statistical comparison between the groups. It does however highlight the long anticipated life expectancy for men with this stage of prostate cancer. Data so far has shown that 7-year cumulative rates of metastatic prostate cancer are reduced in the combined radiotherapy and bicalutamide arm (7% versus 13%; *P* < 0.041) and the rate of freedom from biochemical progression is greater in the bicalutamide arm (57 versus 40%; *P* < 0.0001) with most significant benefit for concomitant bicalutamide seen for those men with gleason grade ≥ 8 (56% and 26%; *P* < 0.0008).

## 5. Prophylactic Breast Bud Radiotherapy and Alternatives

The efficacy of prophylactic breast bud radiotherapy in the prevention of gynaecomastia and breast pain in patients being treated with bicalutamide has been evaluated in a randomised, sham-controlled double-blind trial [6]. 106 patients were randomised to receive a 10 Gy single fraction of breast bud radiotherapy or sham radiotherapy prior to commencing bicalutamide 150 mg daily. A reduction in both investigator and patient assessed gynaecomastia was demonstrated in favour of the prophylactic radiotherapy group (51.9% versus 85.2%; *P* < 0.001; 50% versus 81%; *P* < 0.01). There was a small decrease in breast pain in the radiotherapy group but this did not reach statistical significance. Acute toxicities were transient and well tolerated. Late effects were not accounted for. Although this study showed a modest but significant reduction in breast swelling, half of the men treated with radiotherapy still complained of a degree of breast swelling, and there was no significant reduction in mastalgia.

Alternative treatments have been investigated as prophylaxis for these patients. These include exploiting the anti-estrogenic effects of drugs like tamoxifen, although it is not licensed for this indication. One multicentre prospective trial [7] randomised postprostatectomy patients between bicalutamide, bicalutamide plus tamoxifen, and bicalutamide plus breast bud radiotherapy (12 Gy/1#). Patients in the first group who developed gynaecomastia or mastalgia were then further randomised to receive tamoxifen or radiotherapy. This study demonstrated that tamoxifen was more effective than radiotherapy at preventing and treating gynaecomastia and breast pain, and there was no associated reduction in quality of life, erectile dysfunction, or PSA relapse. In another study, Boccardo et al. [8] randomised 114 patients to receive placebo, tamoxifen, or anastrozole prior to bicalutamide 150 mg therapy. They reported a significant reduction in gynaecomastia in the tamoxifen group, but not the anastrozole group (73% gynaecomastia in control group, 10% tamoxifen, 51% anastrozole *P* < 0.001); a significant reduction in breast pain in the tamoxifen group only (39% versus 6%)—again no significant difference was seen in the anastrozole group. There was no significant difference between groups in those achieving a >50% PSA reduction or for serious adverse events, quality of life, libido, and sexual function.

## 6. Conclusion

In conclusion, we have presented a case where a patient has developed a secondary malignancy following radiotherapy for a benign condition, as prophylaxis against gynaecomastia as a result of bicalutamide therapy. Whilst the indication for breast bud radiotherapy for patients taking stilboestrol in the palliative setting can be supported, the routine use of radiotherapy for patients taking bicalutamide should be disputed. Bicalutamide is often prescribed for younger men in the adjuvant setting or as monotherapy for locally advanced disease. These patients often have a long life expectancy and with continued followup, we would anticipate the risks for developing a second malignancy would increase. We believe that the recommendation for prophylactic breast radiotherapy should be reviewed by NICE and alternative forms of prophylaxis should be considered.
